# Inorganic Reactive Sulfur-Nitrogen Species: Intricate Release Mechanisms or Cacophony in Yellow, Blue and Red?

**DOI:** 10.3390/antiox6010014

**Published:** 2017-02-15

**Authors:** Marian Grman, Muhammad Jawad Nasim, Roman Leontiev, Anton Misak, Veronika Jakusova, Karol Ondrias, Claus Jacob

**Affiliations:** 1 Institute of Clinical and Translational Research, Biomedical Research Center, Slovak Academy of Sciences, Dubravska Cesta 9, Bratislava 845 05, Slovakia; anton.misak@savba.sk (A.M.); veronika.jakusova@gmail.com (V.J.); karol.ondrias@savba.sk (K.O.); 2 Institute of Molecular Physiology and Genetics, Slovak Academy of Sciences, Dubravska Cesta 9, Bratislava 840 05, Slovakia; 3 Division of Bioorganic Chemistry, School of Pharmacy, University of Saarland, Campus B2 1, Saarbruecken D-66123, Germany; jawad.nasim@uni-saarland.de (M.J.N.); roman.leontiev@uni-saarland.de (R.L.)

**Keywords:** cellular thiolstat, diallylsulfanes, nitric oxide, *S*-nitrosothiols, polysulfides, reactive sulfur species

## Abstract

Since the heydays of Reactive Sulfur Species (RSS) research during the first decade of the Millennium, numerous sulfur species involved in cellular regulation and signalling have been discovered. Yet despite the general predominance of organic species in organisms, recent years have also seen the emergence of inorganic reactive sulfur species, ranging from inorganic polysulfides (HS_x_^−^/S_x_^2−^) to thionitrous acid (HSNO) and nitrosopersulfide (SSNO^−^). These inorganic species engage in a complex interplay of reactions in vitro and possibly also in vivo. Employing a combination of spectrophotometry and sulfide assays, we have investigated the role of polysulfanes from garlic during the release of nitric oxide (^•^NO) from S-nitrosoglutathione (GSNO) in the absence and presence of thiol reducing agents. Our studies reveal a distinct enhancement of GSNO decomposition by compounds such as diallyltrisulfane, which is most pronounced in the presence of cysteine and glutathione and presumably proceeds via the initial release of an inorganic mono- or polysulfides, i.e., hydrogen sulfide (H_2_S) or HS_x_^−^, from the organic polysulfane. Albeit being of a preliminary nature, our spectrophotometric data also reveals a complicated underlying mechanism which appears to involve transient species such as SSNO^−^. Eventually, more in depth studies are required to further explore the underlying chemistry and wider biological and nutritional implications of this interplay between edible garlic compounds, reductive activation, inorganic polysulfides and their interplay with ^•^NO storage and release.

## 1. Introduction

The field of sulfur redox biology has changed dramatically since the turn of the Millennium. Centred traditionally around thiols, cysteine, glutathione and a couple of exotic sulfur-containing secondary metabolites found in plants and in fungi, it has now moved on to highlight unusual sulfur modifications and their impact on biological systems. In human biology, posttranslational cysteine and methionine modifications have paved the way for extensive sulfur-centred redox signalling pathways and concepts such as the sulfenome and the cellular thiolstat—concepts always accompanied by widespread hunting and mining activities for unusual yet important sulfur modifications [1,2]. In the field of secondary metabolites, numerous exotic substances have been identified and isolated, not only from plants and fungi but also increasingly from bacteria. Some of these compounds can be seen as “Reactive Sulfur Species” (RSS) in their own right. As selective modifiers of cysteine proteins and enzymes, they may also be “useful” in the development of natural drugs and phytoprotectants. At the same time, sulfur in its various forms has also become a key element in the field of nutrition, with sulfur-based antioxidants and redox-modulators from edible plants such as garlic, onions, mustard and broccoli abundant on any healthy menu. Because of limited bioavailability, high reactivity and sometimes also chemical instability, one may question if those consumable RSS can play a major role inside the human body; yet surely they end up in the gastrointestinal tract where they interact rather strongly with the gut microbiota—and vice versa. Indeed, it seems that today, such organic RSS are omnipresent, from traditional redox biology in cells to plant metabolites, nutrition, drug development and innovation for natural, “green” phytoprotectants based on natural forms of the yellow element.

From a more chemical perspective, the incredible rise of sulfur redox biology witnessed during the last decades has involved numerous rather exotic sulfur species, organic as well as inorganic. Indeed, the last couple of years have seen a true firework of simple inorganic RSS, lighting up a rather dark and mysterious sky in bright yellow, red and also blue. As with a real firework, the chemical species involved are probably short lived, hard to capture and certainly astonishing—yet also highly controversial. Often, it remains a mystery of where they come from, what they do and, after the smoke has cleared, where they have gone.

As part of our own contribution to this 15th anniversary special issue on “Reactive Sulfur Species”, we will try to clear some of this smoke and to show that inorganic RSS are not just a colourful firework of a few elements. They are firmly rooted in sulfur (bio-)chemistry and interconnected with organic RSS, reactive nitrogen species (RNS) and the two main gaseous signalling molecules hydrogen sulfide (H_2_S) and nitric oxide (^•^NO). Furthermore, such inorganic RSS are also involved in major aspects of cellular redox signalling pathways. Here, we will focus on the interplay of metabolites of garlic (mainly diallylsulfanes), first with cellular thiols—a process that leads to the release of H_2_S—and subsequently with *S*-nitrosothiols (via H_2_S) and the ^•^NO signalling pathway.

From the outset, we should emphasize that our studies have been conducted in vitro in order to postulate and map out the *potential* interactions of diallylsulfanes with biological systems and that our findings obviously need to be corroborated in more complex, biological systems. Still, these simple kinetic studies performed by UV/VIS spectroscopy represent the kind of detective ground-work that provides the clue for more eloquent cell based or in vivo investigations.

## 2. Materials and Methods

### 2.1. Chemicals

Organic polysulfanes were synthesized by Muhammad Jawad Nasim at the Jacob’s laboratory (Division of Bioorganic Chemistry, University of Saarland, Saarbruecken, Germany) according to the literature [3,4]. All other chemicals were purchased from Sigma-Aldrich Chemie GmbH (Steinheim, Germany) and used without further purification. Ultrapure water with a resistance ≥ 18 MΩ·cm was generated using a Milli-Q system (Millipore, Darmstadt, Germany). All experiments were performed at room temperature (23 ± 1 °C). Stock solutions of organic polysulfanes were prepared as 10 mM solutions in 200 mM Tris/HCl buffer, pH 7.4 (based on mass and adequate buffer volume). These mixtures were subjected to vortexing and sonication. Samples were stored at −80 °C until use.

### 2.2. UV/VIS Spectroscopy

BRAND^®^ UV micro cuvettes were used in all experiments. For kinetic measurements, an absorption diode array spectrophotometer HP 8452A was employed while for Methylene Blue experiments, a Shimadzu UV-1800 double beam absorption spectrophotometer was used.

### 2.3. Determination of Sulfide Release from Organic Polysulfanes by the Methylene Blue Method

200 µM of organic polysulfanes were incubated with 800 µM of cysteine (Cys)/reduced glutathione (GSH) in 200 mM of Tris/HCl buffer containing 100 μM of diethylenetriaminepentaacetic acid (DTPA), pH 7.4, in plastic microtubes (with minimal space above the solution to avoid evaporation of H_2_S). The reaction mixtures were kept in the dark. After 10 min of incubation at RT, 56 µL of deionized water and 50 µL of 1.88 mM NH_4_OH were added into 394 µL of reaction mixture (final pH ~9.3). This was followed by the addition of a home-made Methylene Blue assay kit which consisted of 250 μL of 54.7 mM zinc acetate, 133 μL of 31.6 mM *N*,*N*-dimethyl-phenylenediamine sulfate (DPD) in 7.2 M HCl and 133 μL of 18 mM FeCl_3_·6H_2_O in 1.2 M HCl. After 15 min of incubation at room temperature, the sulfide content was quantified spectrophotometrically at the wavelength of 668 nm with the aid of a calibration curve that was constructed using authentic sulfide standards (prepared from Na_2_S·9H_2_O) [5]. It should be noted that the presence of Cys and GSH on its own (negative control) exhibited only a minimal impact on the assay.

## 3. Results and Discussion

### 3.1. Selection and Acquisition of Sulfur Compounds

**Figure 1:**
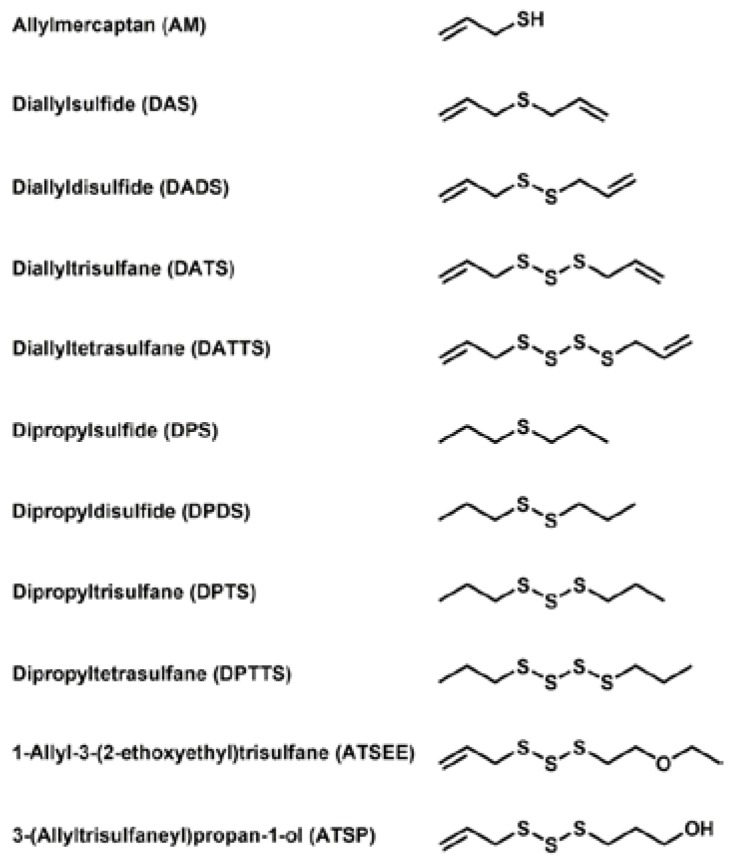
Names, abbreviations and chemical structures of the various sulfur compounds selected for this study. These compounds include a set of different diallylsulfanes found in garlic, dipropylsulfanes found in onions, a reduced mercaptane as a reference and two synthetic derivatives with higher stability, solubility and less odour. It should be noted that the use of the nomenclature of organic and inorganic polysulfide/polysulfane species is still somewhat confusing. In line with the common “taxonomy” of negatively charged anions and hydrogen sulfides, we will therefore for the time being use the expression “sulfide” and polysulfide for anionic, inorganic species of the composition HS_x_^−^/S_x_^2−^, and the expression “sulfane” for organic, uncharged molecules RS_x_R’, in analogy to “alkane”.

In order to comprehend the interaction of natural sulfur-containing compounds, namely diallyl polysulfanes and dipropyl polysulfanes from plants such as garlic and onion, with other cellular species, we have selected a range of relevant and structurally related sulfur compounds for our study. The chemical structures of these compounds are shown in Figure 1. It should be emphasized that these compounds differ in (a) the number of sulfur atoms forming the (poly)sulfane motif; (b) the saturation of the side-chain and (c) the oxidation state, especially with respect to reduced allylmercaptane (AM). Our selection also includes two non-natural compounds which have been designed for better bioavailability and lower volatility and hence odour. Whilst some of the compounds are available commercially, most of them have been synthesized according to the literature or by methods developed recently in our laboratory [6,7].

### 3.2. Nitric Oxide Release from S-Nitrosoglutathione Is Triggered by Reduction of Polysulfanes and Hydrogen Sulfide Formation

The first question posed as part of this study concerns the ability of diallylsulfanes to release ^•^NO from *S*-nitrosoglutathione (GSNO). There have been various reports on a potential ^•^NO release from RSNO triggered by such sulfur species [8,9,10,11,12,13]. Yet from a chemical perspective, such a release would require a nucleophilic attack, and it seems highly unlikely that a mono-, di- or polysulfanes can perform such an attack independently.

**Figure 2:**
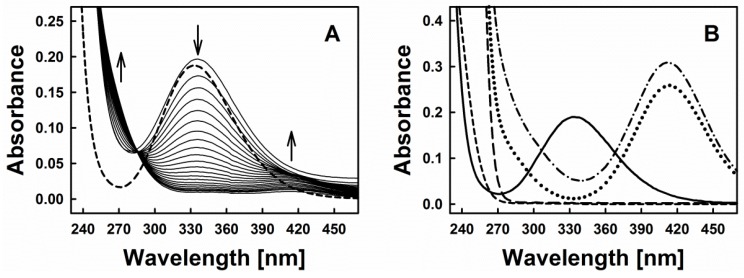
(**A**) Time resolved absorption spectra of 200 µM GSNO (dashed line) and after addition of 200 µM DATS and 800 µM Cys (spectra were recorded every 30 s); (**B**) Absorption spectrum of 200 µM GSNO (solid line); 2 mM H_2_S (prepared from Na_2_S·9 H_2_O, long-dashed line); 2 mM DTT (short-dashed line); spectrum of the mixture of 200 µM GSNO with 2 mM H_2_S after 5 min of their interaction (dash-dotted line) and spectrum of the same mixture treated 10 min with 2 mM DTT (dotted line).

Figure 2A represents time-resolved absorption spectra indicative of the interaction of diallyltrisulfane (DATS) with GSNO and Cys. Based on the typical spectra of GSNO itself or its interaction with excess of sulfide (Figure 2B), we decided to investigate the spectral changes at 270, 334 and 412 nm, which were assigned to the formation of inorganic polysulfides or organic hydroper- and polysulfides (270 nm), decomposition of GSNO (334 nm, ʎ_max_(GSNO)) and formation of the nitrosopersulfide (perthionitrite, SSNO^−^) anion (a rather colourful yellow species with ʎ_max_ at 412 nm) [10,13,14]. It should be mentioned that inorganic per- and polysulfides, as sulfane-sulfur species, show a typical broad absorption spectrum in the range of 260–420 nm (as reported in the literature, e.g., [15]). We intentionally chose 270 nm due to an absorption minimum of GSNO at that wavelength. It should also be noted that also other possible interaction products may contribute to this absorption signal, still we presume that changes at this specific wavelength primarily reflect the formation of inorganic polysulfides or organic hydroper- and polysulfides. Here, our assumption is based on a comparable absorbance of SSNO^−^ at 270 nm and 412 nm (Figure 2B—dotted line, experiment with dithiothreitol (DTT)) and on the fact that in our experiments the absorbance at 412 nm is significantly smaller when compared to the absorbance at 270 nm (~0.01 vs. ~0.06). We have also performed spectral corrections to the light scattering of the DATS sample (A_510 nm_), the contribution of absorbance of polysulfides at 334 nm and the GSNO absorbance at 412 nm. Eventually, kinetic traces at 270 nm reflect the changes of absorbance at this wavelength after addition of Cys to the mixture of GSNO plus organic polysulfanes, kinetic traces at 334 nm reflect changes due to GSNO decomposition and kinetic traces at 412 nm indicate the predicted absorbance of SSNO^−^ formed during the reaction.

**Figure 3:**
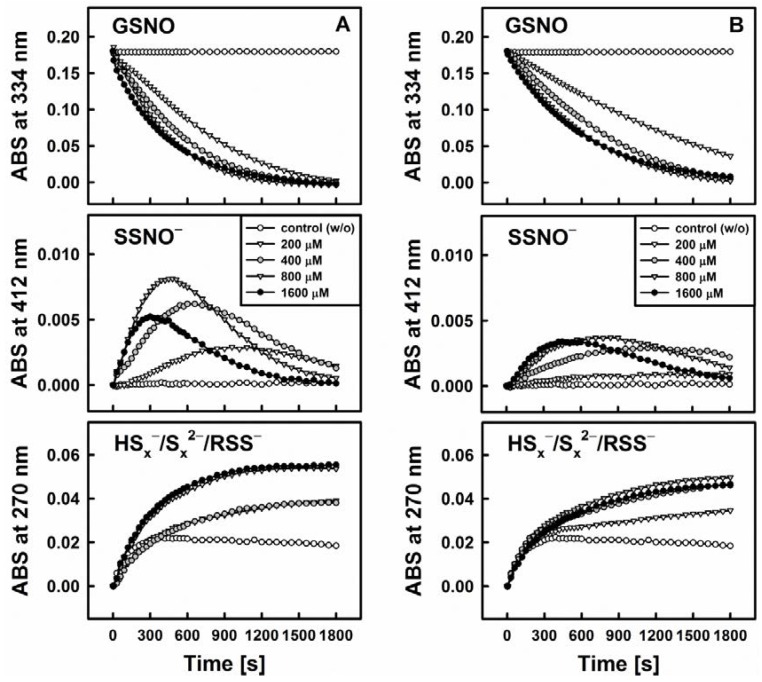
Kinetic traces of the interactions of 200 µM DATS with 200 µM GSNO in the absence and presence of various concentrations of cysteine (Cys, left, **A**) and reduced glutathione (GSH, right, **B**) monitored at three characteristic wavelengths by UV/VIS spectrophotometry (334 nm—GSNO decomposition, 412 nm—formation of SSNO^−^, 270 nm—formation of hydroper-/polysulfides). The maximum rate of GSNO decomposition was achieved at concentrations of 800 or 1600 µM Cys/GSH.

Figure 3 exhibits the representative example of DATS which indeed is unable to decompose GSNO single-handedly on its own. Interestingly, when a thiol-based reducing agent, such as Cys or GSH, is added, the system becomes “activated” in a concentration dependent manner and GSNO is decomposed rapidly, i.e., within 30 min, as can be observed by a sharp decline in the absorbance at 334 nm (ʎ_max_(GSNO)).

One of the crucial findings of our study is that this sequence of reactions requires the presence of all three components, i.e., organic polysulfane, thiol and GSNO. The decomposition of GSNO and formation of SSNO^−^ is dependent on the presence of thiols (such as Cys and GSH) in the reaction system, where they serve as activators of the organic polysulfanes. On their own, however, these thiols do not decompose GSNO or SSNO^−^. SSNO^−^ is resistant toward a range of thiols (Cys, GSH, DTT) and even to potassium cyanide (KCN) [10], but it is not resistant towards the commonly used phosphine-based disulfide reductant Tris(2-carboxyethyl)phosphine hydrochloride (TCEP, unpublished results).

Still, the matter is more complicated, and the role of thiols in those assays is probably double-edged. In the presence of H_2_S, GSH slows down the decomposition of GSNO and decreases the yield of SSNO^−^ more considerably compared to Cys. This effect is in accordance with the p*K*_a_ value of thiol groups (p*K*_a_(GSH) > p*K*_a_(Cys)). For instance, the presence of 5 mM of GSH in the mixture of 200 µM GSNO and 200 µM Na_2_S almost completely abolishes the formation of SSNO^−^ [12]. The mechanism by which the presence of reduced and low molecular thiols influences the rate of GSNO decomposition and SSNO^−^ formation is therefore still somewhat controversial. We speculate about their interaction with the persulfide anion HSS^−^ as an intermediate: The latter almost certainly is responsible for the formation of SSNO^−^ and as inorganic disulfide indeed is also sensitive to further reduction by thiols [10].

Eventually, these simple in vitro findings are highly informative and point towards possible mechanism(s) by which natural organic polysulfanes may interact with *S*-nitrosothiols and also ^•^NO release and signalling. It seems, for instance, that the oxidized forms of sulfur, such as di-, tri- and tetrasulfanes, cannot decompose GSNO independently, as has been anticipated, but are activated by thiols to form some intermediate RSS^−^/HSS^−^, including SSNO^−^ which is followed by the formation of polysulfides, persulfides and ^•^NO. Moreover, these findings support previous reports by us and others which have demonstrated that an excess of (inorganic) sulfide reacts with *S*-nitrosothiols and thereby leads to the generation of four major products, namely inorganic polysulfides (HS_x_^−^/S_x_^2−^), nitrosopersulfide (SSNO^−^), dinitrososulfite (or *N*-nitrosohydroxylamine-*N*-sulfonate, SULFI/NO) and nitroxyl (HNO). Beside those major products, a number of other, minor sulfur containing inorganic species, mainly sulfite (as HSO_3_^−^), sulfate (as HSO_4_^−^) and thiosulfate (as HS_2_O_3_^−^) are also produced. The eventual formation of ^•^NO itself is therefore the result of one-electron oxidation of the nitroxyl anion or of homolytic cleavage of SSNO^−^ to the disulfide radical anion (SS^•−^) and ^•^NO [10].

Before we turn our attention to the underlying “chemistry” of such processes, we should add a few words on SSNO^−^, whose formation and stability in aqueous solutions is still under debate (reviewed in [16,17]), and we will turn to possible alternative mechanisms for ^•^NO release later on. In order to provide a balanced view on the subject, it is worth to briefly consider the present arguments. The initial characterization of SSNO^−^ was carried out by Seel et al. in the 1980s [18,19,20,21]. Yet Wedmann et al. showed that SSNO^−^ (prepared as bis(triphenyl)phosphaniminium perthionitrite (PNP^+^SSNO^−^) salt in acetone) is unstable after irradiation (15 s for complete decomposition), addition of water (10% of volume, 10 min for complete decomposition) or acidification with concomitant formation of nitroxyl (HNO) and elemental sulfur. Addition of sulfide also leads to irreversible decomposition of SSNO^−^ in a 10% water-90% acetone mixture. SSNO^−^ is not stabilized by excess of sulfide, and indeed those two sulfur species react together with subsequent formation of SNO^−^, which has been presumed by various authors as a more stable species compared to SSNO^−^ [22].

In the end, SSNO^−^ is neither the smallest nor most obvious inorganic sulfur-nitrogen species, and there have been attempts to hunt down some of its relatives in biology as well. Filipovic et al. have recently shown that thionitrous acid (HSNO), the smallest *S*-nitrosothiol on paper, is a product of the GSNO-H_2_S interaction. It can diffuse across biological membranes and subsequently elicits various physiological responses. Those authors have assigned a product with ʎ_max_ at 412 nm belonging to polysulfide, not to SSNO^−^ [9].

In contrast, formation of SSNO^−^ in water mixture of 1 mM DEA/^•^NO (diethylamine NONOate) or SNAP (*S*-nitroso-*N*-acetyl-DL-penicillamine) with 2 mM Na_2_S has been confirmed by electrospray ionization-high-resolution MS analysis (ESI-HRMS) [10]. Fukuto’s group showed that MCP-SSNO (*N*-methoxycarbonyl penicillamine nitrosopersulfide), as the product of the interaction of GSNO with persulfide MCP-SSH, is unstable and decomposes to MCP-SS^•^ and ^•^NO through homolytical cleavage [23]. Computational analysis showed that the S-N bond in RSS-NO is extremely weak at room temperature (1.85 Å in CH_3_SSNO, 1.953 Å in *cis*-HSSNO and 1.878 Å in *trans*-HSSNO) in comparison to SSNO^−^ (1.73 Å, or 1.676 Å in *cis*-SSNO^−^ and 1.695 Å in *trans*-SSNO^−^) [22,23]. This shortening of S-N bond in the anion SSNO^−^ leads to the partial double bond character of the S-N bond [23]. Another recent computational study, this time by Olabe’s group, points out that SSNO^−^ is the intermediate during the NO-H_2_S “cross-talk” [24]. Pluth et al. showed that SSNO^−^ is also an intermediate of the reaction of isolated persulfides and NO_2_^−^ and it is stable upon addition of water [25]. In the end, the unexpected stability of SSNO^−^ seems to be the result of charge delocalisation, which, of course, is not possible for SNO^−^ [17].

In any case, sequence of following reactions accounts for the observations of our study, i.e., the decomposition of GSNO, the formation of SSNO^−^ and the liberation of ^•^NO [10]:

RSNO + HS^−^ → RS^−^ + HSNO

HSNO + HS^−^ → HSS^−^ + HNO (HSSH + NO^−^)

HSS^−^ + RSNO → SSNO^−^ + RSH

HSS^−^ + HSNO → SSNO^−^ + H_2_S (HS^−^ + H^+^)

SSNO^−^ → SS^•−^ + ^•^NO

The appearance of inorganic polysulfides can be explained by a set of “follow-on” reactions [26,27]:

2 HS_2_^−^ ↔ HS_3_^−^ + HS^−^

HS_2_^−^ + HS_3_^−^ ↔ HS_4_^−^ + HS^−^

HS_3_^−^ + HS_3_^−^ ↔ HS_4_^−^ + HS_2_^−^

HS_4_^−^ + HS_2_^−^ ↔ HS_5_^−^ + HS^−^

2 SS^•−^ → S_4_^2^^−^ → S^•−^ + S_3_^•−^

HS_(*n* + 1)_^−^ + H^+^ → *n*/8 S_8_↓ + H_2_S (*n* = 1–8)

According to Giggenbach and Kamyshny et al. the disulfide ion is the predominant species only at extremely high alkaline pH. The pentasulfide ion is formed in significant amounts only in nearly neutral or slightly acidic solutions [28,29].

Nonetheless, this sequence of reactions immediately raises the question which species are responsible for the attack on GSNO, as we have added only organic sulfanes and thiols but no sulfides or H_2_S. Here, the initial finding—that none of the DATS, Cys or GSH release ^•^NO from GSNO when used alone, but seem to act in concert—provides a lead. It is known that this kind of interaction results in the formation of allylmercaptane (AM), a couple of allyl persulfides such as allyl-SS^−^ and, notably, H_2_S, predominantly in the form of HS^−^ [30]. Among those species formed, AM on its own is unable to decompose GSNO, and does not become “activated” in the presence of a reducing thiol either. It therefore appears that the “active species” in the DATS interaction with GSNO is indeed an inorganic sulfide, such as HS^−^.

**Figure 4:**
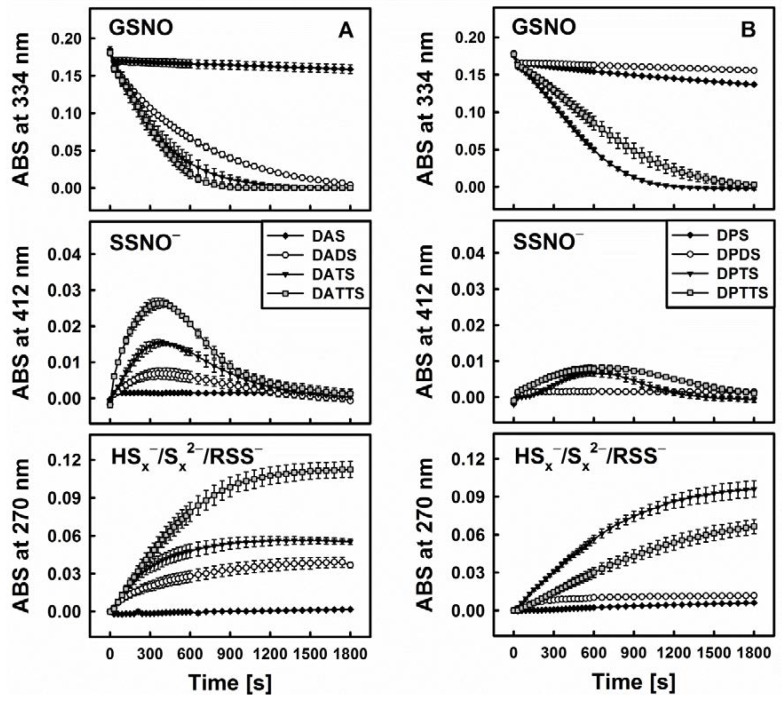
Kinetic traces of the interactions of sulfanes (200 µM) of different sulfur-sulfur chain lengths (from 1 to 4) with GSNO in the presence of 800 µM Cys monitored at three characteristic wavelengths by UV/VIS spectrophotometry. Allyl compounds characteristic of garlic (**A**) and propyl analogues characteristic of onions (**B**) (*n* = 3).

This hypothesis is corroborated by several additional findings. First of all, when comparing DATS to its three “relatives”, namely diallylsulfide (DAS), diallyldisulfide (DADS) and diallyltetrasulfane (DATTS), there are certain kinetic similarities between the ability of GSNO decomposition on the one side and H_2_S release on the other (Figure 4). As these organic polysulfanes are known to release inorganic sulfides, the involvement of such species can be expected. Still, *S*-nitrosothiols, e.g., GSNO, are also electrophilic nitrosating agents [31]. Due to the fact that persulfides (RSS^−^) are strong nucleophiles [32], a mutual interaction involving these two species—rather than inorganic sulfides (HS_x_^−^) can lead to RSS-NO formation with subsequent homolytic cleavage to RSS^•^ + ^•^NO [23]. In any case, “size matters” as far as the sulfur-chain in the diallylsulfanes and dipropylsulfanes is concerned (Figure 4).

Within this context, DAS, being a monosulfide, cannot be transformed by Cys or GSH to a thiol or H_2_S. It is also unable to trigger the decomposition of GSNO. In contrast, DADS is readily reduced to AM in the presence of thiols, but it is only a weak donor of H_2_S, which is in line with its lower reactivity towards GSNO. It is, therefore, unlikely that AM is the active species involved in the decomposition of GSNO. In contrast, DATTS, in the presence of thiols, is an excellent H_2_S releasing agent and under those conditions also reacts readily with GSNO. Interestingly, the tetrasulfane, when compared to the trisulfane, is able to form a range of additional RSS, such as organic persulfides (allyl-SS^−^) and inorganic HS_x_^−^/S_x_^2−^. Yet in our studies the activity of tetrasulfane was not significantly different from the one of trisulfane. In case of allylic compounds, tetrasulfane was more active than trisulfane while in case of the propylic compounds it was vice versa. Despite its additional chemical possibilities, the tetrasulfane is not significantly more active as compared to the trisulfane, supporting the notion that a simple inorganic sulfide, rather than mercaptanes, persulfides or fancy inorganic di- or polysulfides may be at the centre of such interactions.

**Figure 5:**
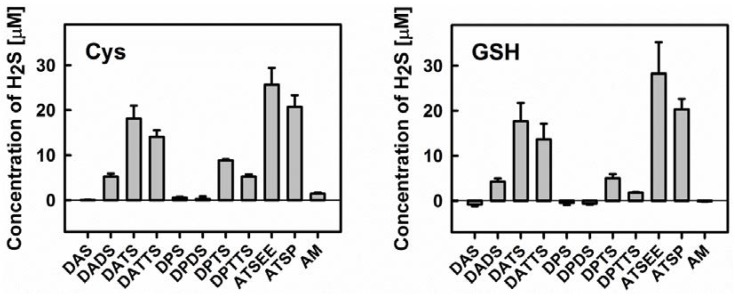
**Left:** Release of H_2_S from different organic (poly)sulfanes in the presence of cysteine (Cys). **Right:** Release of H_2_S from different organic (poly)sulfanes in the presence of reduced glutathione (GSH). Concentrations of H_2_S were quantified via the Methylene Blue method after 10 min of incubation with 800 µM Cys/GSH and 200 µM organic (poly)sulfanes (*n* = 3).

Secondly, we have employed the Methylene Blue method to determine H_2_S release from the various organic sulfanes in the absence and presence of cysteine or GSH. The results shown in Figure 5 are in excellent agreement with previous literature studies on diallylsulfanes and support our initial hypothesis regarding their interaction(s) with GSNO [30,33,34]. Both, cysteine and GSH are able to release H_2_S from the various organic sulfanes. The extent of H_2_S release from different substances agrees well with the trend observed for GSNO decomposition. Neither DAS nor its propyl analogue dipropylsulfide (DPS) (and also dipropyldisulfide DPDS) can release any H_2_S. The same applies to AM—ruling out a possible role of this nucleophile as active species—and also applies to Cys or GSH. In contrast, the disulfide DADS can release some H_2_S, yet in considerably less amount when compared to the respective trisulfanes DATS and dipropyltrisulfane (DPTS). Interestingly, the tetrasulfanes DATTS and dipropyltetrasulfane (DPTTS) are surprisingly poor H_2_S donors, which could be attributed either to their inherent chemical instability or to the formation of additional sulfur species which ultimately compete with the release of H_2_S (see above).

None of the compounds were able to release H_2_S when used alone (data not shown). It is in agreement with results of DeLeon et al. who, in an assay based on the H_2_S-sensitive fluorescent dye 7-azido-4-methylcoumarin (AzMC), demonstrated that garlic oil and DATS, when used independently, cannot release H_2_S [35]. Interestingly, normoxic and hypoxic conditions showed comparable effects on H_2_S-release from garlic oil and DATS in the presence of Cys or GSH, jettisoning the idea of a crucial involvement of dioxygen.

Results of Liang et al. indicated a mechanism of interaction of GSH with DADS and DATS. DADS rapidly reacts with GSH via thiol-disulfide exchange to generate AM and GSSA (S-allyl glutathione disulfide). GSSA reacts with another GSH and produces of AM and GSSG. This mechanism does not include the generation of H_2_S (contrary to work of Benavides et al. [30]). Then again, they proposed two possible routes of the interaction between DATS and GSH. The first one includes the generation of GSSA and ASSH, which reacts with GSH to release H_2_S. The second one includes the generation of AM and GSSSA and in next steps the generation of GS_n_A and AS_n_A. When GSH is in excess over DATS, H_2_S may well be released from GS_n_A and AS_n_A [36].

### 3.3. Smell Another Day: Synthetic Polysulfanes as a more Practical Alternative?

In the previous section it has already been mentioned that the specific (poly)sulfur-motif found in different organic polysulfanes is crucial for their ability to release H_2_S (and other inorganic HS_x_^−^/S_x_^2−^ species) upon reduction with common cellular thiols. Indeed, this notion holds true for mono-, di- and trisulfanes. Whilst compounds such as AM, DAS, DPS and DPDS cannot release H_2_S and hence are also unable to interact with GSNO, the ability to interact increases significantly when moving from the mono- to the di- and then to the trisulfanes. The latter exhibit the maximum activity and even tetrasulfanes do not seem to have a significantly stronger impact on GSNO.

Therefore trisulfanes seem to provide an acceptable compromise between stability on the one side and reactivity on the other—and may even be considered as natural H_2_S or ^•^NO releasing agents. Nonetheless, both, DATS and DPTS are difficult to apply in practice due to their oily consistency and poor solubility. Furthermore, their pungent smell also prevents any widespread practical applications outside Gaul, for instance in medicine or in agriculture.

We have, therefore, included two synthetic trisulfanes in our study, which represent potential alternatives to DATS or DPTS. Those asymmetric trisulfanes feature the allyl group on the one side of the trisulfane chain whilst on the other side, they contain a group that increases stability and reduces volatility. As shown already in Figure 5, both, the alcohol ATSP (3-(allyltrisulfaneyl)propan-1-ol) and the ether ATSEE (1-allyl-3-(2-ethoxyethyl)trisulfane) are able to release H_2_S upon reduction by cysteine or GSH. ATSEE seems to be particularly potent in this Methylene Blue assay i.e., with H_2_S release almost 1.5-fold higher compared to the natural trisulfanes DATS or DPTS.

**Figure 6:**
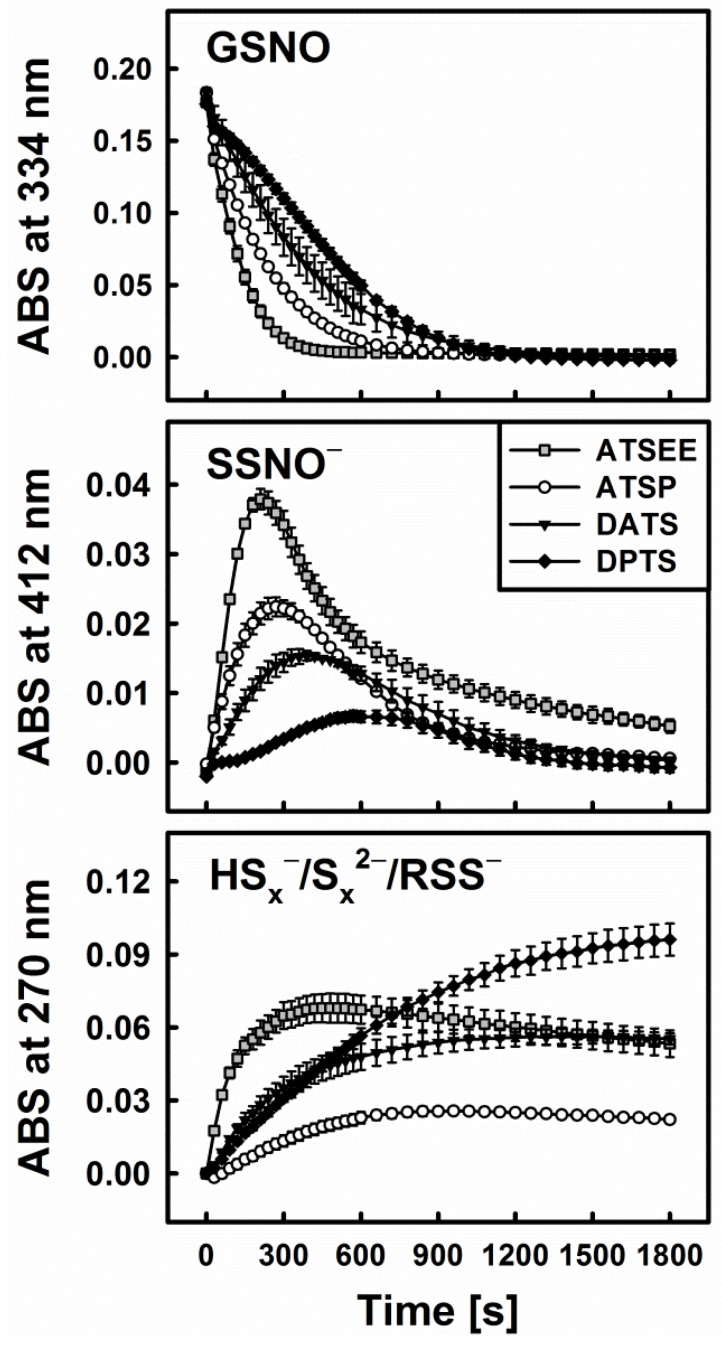
Kinetic traces of the interactions of 200 µM of synthetic trisulfanes ATSP and ATSEE, respectively, with 200 µM GSNO in the presence of 800 µM Cys. The traces for DATS and DPTS are shown for comparison.

In line with these observations and our hypothesis, i.e., that reduction releases H_2_S from organic polysulfanes and therefore explains their ability to decompose GSNO, a strong interaction of ATSP and ATSEE with GSNO in the presence of cysteine or GSH has also been noticed. Figure 6 illustrates this superior behaviour of the synthetic trisulfanes. ATSEE, in particular, readily and rapidly decomposes GSNO once “activated” via reduction with cysteine. It also causes the temporary appearance of the characteristic yellow colour of SSNO^−^, followed by a rapid build-up of disulfide species. ATSP is slightly less active, in line with its somewhat lesser ability to liberate H_2_S upon reduction (see Figure 5).

### 3.4. Polysulfanes, H_2_S, H_2_S_x_, GSNO and ^•^NO: Complex Interactions or Cacophony?

We will now turn our attention to a more mechanistic and to some extent also more speculative discussion. When considering the interactions between di- and polysulfanes, cysteine or GSH, and GSNO, the results obtained as part of the in vitro studies point towards a sequence of chemical reactions, which among others involves H_2_S release, the formation of SSNO^−^ and the eventual appearance of certain disulfides. We have already proposed such reactions above, yet primarily in a simplified in vitro scenario. Let’s therefore briefly consider some more intricate questions disseminating from such a simple scheme based on—comparably simple—spectroscopic observations.

RSNO + HS^−^ → RS^−^ + HSNO

HSNO + HS^−^→ HSS^−^ + HNO (HSSH + NO^−^)

HSS^−^ + RSNO → SSNO^−^ + RSH

HSS^−^ + HSNO → SSNO^−^ + H_2_S (HS^−^ + H^+^)

This more traditional sequence of reactions proposed for the liberation of NO^−^ from GSNO in the presence of H_2_S—and eventually the formation of ^•^NO, involves several steps which require the correct interaction of RSS with various partners [10]. Whilst some of the species observed in our studies are also eventually formed as part of this scheme, the latter appears to be rather complex. In biological systems, the reaction partners are present in orders of magnitude lower concentrations than in our in vitro experiments, and it therefore needs to be shown if the reactions envisaged actually transcend into the biological sphere or are simply a piece of good chemistry.

As mentioned already, the reductive “activation” of organic polysulfanes such as DATS results in the formation of organic RSS^−^ species which in turn may attack GSNO to form a short-lived intermediate RSS-NO which rapidly breaks up to liberate RSS^•^ and ^•^NO.

RSS^−^ + GSNO + H^+^ → RSS-NO + GSH

RSS-NO → RSS^•^ + ^•^NO

Compared to the traditional sequence of events, this mechanism may provide a comparably simple explanation for GSNO decomposition and ^•^NO release in the presence of—partially—reduced organic persulfides (RSS^−^). It accounts for the fact that three components, namely GSNO, a polysulfane and a thiol are required for GSNO decomposition, and assigns a pivotal role to the organic perthiol species, which is clearly more nucleophilic and hence reactive than a simple thiol, such as cysteine, GSH and AM (the latter is also formed but inactive when applied in respective controls). This two-step mechanism cannot be corroborated easily because the intermediate RSS-NO cannot be detected in the absorption spectra due to its quick decomposition and unspecific spectroscopic properties. Still, there is some evidence in the literature which supports this mechanism, at least in theory [23,25]. The second reaction, for instance, is not reversible, as results of Fukuto’s group show that RSS^•^ does not appear to react with ^•^NO [23].

Alternatively, GSNO offers two possible sites for a nucleophilic attack, not only at the nitrogen, but also at the less electronegative sulfur atom. If one toys with the idea of an attack of H_2_S at the sulfur, then a direct release of NO^−^ with concomitant formation of a GSS_x_^−^ species via an intermediate GS(S)NO^−^ species would be feasible.

HS_x_^−^ + GSNO → GSS_x_^−^ + NO^−^ + H^+^

(RSS^−^ + GSNO → RSSSG + NO^−^)

This pathway may also account for some of our observations, such as loss of GSNO, appearance of a SSNO^−^-like structure and subsequent formation of a disulfide. Despite the fact that it is less complex compared to reaction sequence discussed before, it is equally uncertain if it actually occurs in a complicated biological system.

### 3.5. Biological Relevance of the Diallyltrisulfane, Thiol and GSNO Ménage à Trois: Hard to Get or Getting Hard?

Considering the various caveats, complications and debates associated inherently with the interaction of organic sulfanes, thiols, GSNO and ^•^NO in vitro, it is hardly surprising that there is also an argument to which extent this “chemistry” is relevant in biology, if at all. Some of us have recently reported that products of H_2_S-GSNO interaction, probably SSNO^−^ and HNO, show a pronounced impact on aortic ring relaxation in comparison to GSNO [37]. These species also decrease blood pressure in rats [10], activate soluble guanylyl cyclase [13] and trigger the Keap-1/Nrf2 redox system [38].

Comparable biological effects have been observed for garlic extracts and/or garlic-derived polysulfanes. An aqueous garlic extract, for instance, results in a prolonged relaxation of aortic rings in vitro [33] and decreased blood pressure [39]. As those physiological outcomes are typically related to the action of ^•^NO, these findings may point toward a connection between garlic products and ^•^NO via nitroso-compounds.

Nonetheless, there are certain issues with this line of argument, especially in the context of concentrations and the emerging physiological “rivalry” between ^•^NO and H_2_S. The concentrations of H_2_S reported in blood and tissues vary from tens of nanomolars up to tens of micromolars, or even up to 0.2–3.4 mM in the luminal content of the large intestine [40,41,42]. High concentrations of H_2_S in the colon show a prolonged effect on rat blood pressure [43], which could be also achieved by garlic consumption, hence providing a direct link between these two sulfur species without the necessity of ^•^NO or ^•^NO release [33].

Indeed, the concentration of *S*-nitrosothiols in tissues varies from ten to hundreds of nanomolar [44]. H_2_S is therefore at 10–100 excess over S-nitrosothiols. The rate constant for SSNO^−^ formation (from the reaction of sulfide with GSNO at the ratio 2:1) has been calculated as 640 ± 200 M^−1^·s^−1^ [45] and for HSNO formation (1:1 ratio) as 84 ± 7 M^−1^·s^−1^ [9]. At the same time, the rate constant for the interaction of nitroprusside and HSNO with HS_2_^−^ is ~100-fold higher than with HS^−^ [24,46]. Using the results of pulse radiolysis from Filipovic et al. [9], Koppenol and Bounds have therefore estimated the rate constant of the reaction between HSNO and HS^−^ (leading to formation of HSS^−^ and HNO) to be 10^7^ M^−1^·s^−1^. Based on physiological concentrations of ^•^NO and H_2_S and their mutual interaction, they have also predicted that the rate of SSNO^−^ formation could be only ~10^−14^ M^−1^·s^−1^ [47]. Together, these kinetic parameters indicate that SSNO^−^ production under such conditions is not biologically relevant. Even worse, GSH is quite abundant and occurs in cells, tissues or blood at millimolar concentrations. Since GSH can react with disulfide to form glutathione persulfide and HS^−^, it can further lower the SSNO^−^ yield [12,47].

Therefore SSNO^−^ seems to be hard to get in a well functioning biological system and may only play a role under imbalanced conditions, for instance when the GSH concentration is reduced (e.g., under oxidative stress) or when the concentrations of H_2_S and RSNO are elevated, e.g., in the gut after a major consumption of garlic or after consumption of food with high levels of nitrate [48]. The issues surrounding the formation of SSNO^−^ in Biology are far from trivial and clearly require more attention. Interestingly, Nava et al. have proposed a reaction between ^•^NO and H_2_S in vivo, under anaerobic conditions and without N_2_O_3_ as an intermediate, which may also result in the formation of SSNO^−^ through a different reaction mechanism involving HSNO as an intermediate [10,49].

In any case, and regardless of the precise mechanism or mechanisms ultimately at work, and the exact nature of the chemical species involved, the idea that compounds such as DATS may exert their frequently mentioned biological activity via decomposition of GSNO and interfere with ^•^NO signalling, is interesting, but at the same time provides just a small additional contribution to the colourful mosaic of biological actions associated with such natural trisulfanes. Simply looking at the concentration, reactivity and biological impact, direct oxidative modifications of thiol groups in proteins, enzymes or indeed oxidation of GSH are more likely to occur, and therefore cellular redox homeostasis and signalling pathways of the cellular thiolstat become affected [2,50,51]. As above, the interaction of such organic polysulfanes with GSNO via H_2_S may become only relevant under certain, exceptional conditions, for instance during periods of low peptide or protein thiol content. The question of the exact target or targets of polysulfanes may lead to a variety of answers, depending on the circumstances, and in any case reflects the multiple functions of RSS within a complex biology, whereby such molecules almost certainly infringe on a number of different targets and pathways [52].

As far as “gaseous” signalling involving H_2_S or ^•^NO is concerned, a release from polysulfanes or GSNO, respectively, may not be the most prominent avenue either. There are probably other, often enzymatic systems, which are more effective in generating either H_2_S or ^•^NO. At the same time, there may be considerably more interactions affected when discussing the complex medley of reactive sulfur-centred species which not only include trisulfanes and H_2_S, but also thiolate anions (RS^−^), persulfides (RSS^−^) and, almost unavoidably, some HS_x_^−^/S_x_^2−^ species.

Nonetheless, despite these issues, the studies presented highlight the amazing interactions which are possible in sulfur redox biology and underline once more the need to consider all the RSS, not only a few usual aspects, but also more elusive species from edible plants, organic and, eventually, inorganic chemistry. Any such consideration will be fairly hard as it also has to pay tribute to the various “small molecule interactions”, as RSS often do not act alone, but also interfere with H_2_S and ^•^NO signalling. Here, particular attention needs to be paid to concentrations and kinetic parameters. In other words, wherever there are sulfur species in biology, they will interact with each other, other reactive species and eventually also with their targets, some faster and others more slowly.

## 4. Conclusions

In summary, our in vitro studies have demonstrated that compounds such as DATS and DATTS react readily with a range of biological molecules. Their ability to release significant amounts of H_2_S upon reduction with common thiols, such as cysteine or GSH, also enables them to decompose GSNO rather readily, and hence to also trigger the ^•^NO signalling pathway. Future studies will therefore be required to determine the mode(s) of action of such culinary polysulfanes more carefully, and also with an open mind and open eye for less apparent but possibly equally important avenues. Here, a complicated interplay between different redox and signalling pathways exceeding by far traditional notions of a simple “crosstalk” may have to be considered. Eventually, it is the cell biologist who will have to demonstrate that such interactions indeed occur inside intact cells, that they possess a biological significance and that they accumulate to a true signalling symphony, a trumpet concerto in E flat major of gaseous and non-gaseous molecules, and not simply to a random cacophony of mutually reactive, yet otherwise uncoordinated redox players.

## Abbreviations

GSNO, S-nitrosoglutathione; HS_x_^−^/S_x_^2−^, inorganic polysulfides; SSNO^−^, nitrosopersulfide (perthionitrite); ^•^NO, nitric oxide; H_2_S, hydrogen sulfide; Cys, cysteine; GSH, reduced glutathione; HSNO, thionitrous acid; RSS, reactive sulfur species; RSNO, S-nitrosothiols; HNO, nitroxyl; DTT, dithiothreitol; HSS^−^, persulfide (disulfide) anion; RSS^−^, organic persulfide anion; AM, allylmercaptan; DAS, diallylsulfide; DADS, diallyldisulfide; DATS, diallyltrisulfane; DATTS, diallyltetrasulfane; DPS, dipropylsulfide; DPDS, dipropyldisulfide; DPTS, dipropyltrisulfane; DPTTS, dipropyltetrasulfane; ATSEE, 1-allyl-3-(2-ethoxyethyl)trisulfane; ATSP, 3-(allyltrisulfaneyl)propan-1-ol.
